# Deep Learning Radiomics to Predict Regional Lymph Node Staging for Hilar Cholangiocarcinoma

**DOI:** 10.3389/fonc.2021.721460

**Published:** 2021-10-26

**Authors:** Yubizhuo Wang, Jiayuan Shao, Pan Wang, Lintao Chen, Mingliang Ying, Siyuan Chai, Shijian Ruan, Wuwei Tian, Yongna Cheng, Hongbin Zhang, Xiuming Zhang, Xiangming Wang, Yong Ding, Wenjie Liang, Liming Wu

**Affiliations:** ^1^ Department of Radiology, Yiwu Central Hospital, Yiwu, China; ^2^ Department of Radiology, The First Affiliated Hospital, College of Medicine, Zhejiang University, Hangzhou, China; ^3^ Polytechnic Institute, Zhejiang University, Hangzhou, China; ^4^ Department of Radiology, Jinhua Municipal Central Hospital, Jinhua, China; ^5^ Department of Hepatobiliary and Pancreatic Surgery, The First Affiliated Hospital, Zhejiang University School of Medicine, Hangzhou, China; ^6^ College of Information Science & Electronic Engineering, Zhejiang University, Hangzhou, China; ^7^ Department of Pathology, The First Affiliated Hospital, College of Medicine, Zhejiang University, Hangzhou, China

**Keywords:** radiomics, hilar cholangiocarcinoma, computed tomography, lymph node, deep learning

## Abstract

**Background:**

Our aim was to establish a deep learning radiomics method to preoperatively evaluate regional lymph node (LN) staging for hilar cholangiocarcinoma (HC) patients.

**Methods and Materials:**

Of the 179 enrolled HC patients, 90 were pathologically diagnosed with lymph node metastasis. Quantitative radiomic features and deep learning features were extracted. An LN metastasis status classifier was developed through integrating support vector machine, high-performance deep learning radiomics signature, and three clinical characteristics. An LN metastasis stratification classifier (N1 *vs*. N2) was also proposed with subgroup analysis.

**Results:**

The average areas under the receiver operating characteristic curve (AUCs) of the LN metastasis status classifier reached 0.866 in the training cohort and 0.870 in the external test cohorts. Meanwhile, the LN metastasis stratification classifier performed well in predicting the risk of LN metastasis, with an average AUC of 0.946.

**Conclusions:**

Two classifiers derived from computed tomography images performed well in predicting LN staging in HC and will be reliable evaluation tools to improve decision-making.

## 1 Introduction

Hilar cholangiocarcinoma (HC) is one of the malignant tumors with poor prognosis, accounting for approximately two-thirds of all biliary tract tumors (1, 2). As far as we know, radical surgical resection is the most potential cure for HC patients to achieve long-term survival (3, 4). Studies confirm that lymph node (LN) status is an important biomarker for HC prognosis (5). Meanwhile, the stage of metastatic LNs (N1: one to three; N2: four or more) as a factor of poor prognosis was incorporated into the tumor node metastasis staging system (6). In clinical practice, LN dissection is a critical surgical step for suspicious LN-positive HC (7). However, routine LN dissection does not provide significant therapeutic benefits for LN-negative HC patients because of the increasing incidence of postoperative complications (8). Therefore, precise evaluation of regional LN staging in HC is critical to formulating individualized clinical treatment strategies.

Traditional imaging characteristics are poorly predictive of the accurate evaluation of LN staging in HC (9). Depending on assessment using the naked eye, LN metastasis cannot be well distinguished from benign swollen nodes (10). Although several advanced imaging technologies have certain values for the prediction of LN status, further study is urgently needed to obtain stable indicators. Hence, there is still a lack of reliable tools for evaluating LN staging in HC.

In recent years, the LN status has been proven to be an important indicator in different tumors and has a marked impact on the prognosis of patients (11, 12). Meanwhile, radiomics models have achieved significant efficiency in predicting the LN status of various malignant tumors (13, 14). Two radiomics studies demonstrated that the radiomics signature (RS) was a potential imaging biomarker of LN status prediction for biliary tract cancer and intrahepatic cholangiocarcinoma (15, 16). Therefore, the radiomics method may also be a valuable tool for LN staging in HC. Nevertheless, there are only a few studies on LN evaluation in HC through machine learning or deep learning.

Thus, our study focuses on developing deep learning radiomics models for predicting the LN status and stratifying LN metastasis in HC based on preoperative computed tomography (CT) data. Our radiomics models integrating deep learning features and clinical characteristics will be beneficial for an individualized evaluation of LN staging.

## 2 Methods

### 2.1 Patients

The Institutional Review Boards of both The First Affiliated Hospital, School of Medicine, Zhejiang University (institution I) and JinHua Center Hospital (institution II) accepted our study and renounced the requirement of patients’ informed consent. The imaging dataset contained 179 contrast-enhanced CT images of 179 HC patients (institution I: 158 patients; institution II: 21 patients) continuously collected from January 2011 to October 2019. Each patient in the dataset was imaged once. For institution I, 81 patients were pathologically diagnosed with LN metastasis, while the others were non-metastatic. There were nine metastatic patients in institution II. There were 70 and 20 patients who were confirmed to be stage N1 (one to three metastatic LNs) and N2 (more than three metastatic LNs) in institutions I and II, respectively. The imaging data from institution I were used as the training cohort to train the structure. To improve the confidence of the results, the imaging data from institution II were also used in the study as an external test cohort.

The inclusion criteria were as follows: 1) patients undergoing radical operation, LN dissection, and pathological confirmation; 2) contrast-enhanced CT scans performed within 1 month before surgery; and 3) availability of CT images and pathological specimens. The exclusion criteria were as follows: 1) palliative surgery; 2) poor quality images that cannot be used for delineation; 3) recurrent or metastatic lesions; and 4) antitumor therapy before CT scans. The recruitment pathway for patients is illustrated in **Supplementary Figure S1**.

### 2.2 Clinical Characteristics

Clinical characteristics such as age, sex, maximum tumor diameter, preoperative plasma carcinoembryonic antigen (CEA) level (positive or negative), carbohydrate antigen 19-9 (CA 19-9) level (positive or negative), and clinical stage were derived from electronic medical records. The specific thresholds distinguishing the levels (positive or negative) of two indicators mentioned above were 5.0 ng/ml (CEA) and 37.0 U/ml (CA 19-9).

### 2.3 Image Acquisition

An Aquilion CT scanner (16-slice; Toshiba Medical Systems, Tokyo, Japan) and a Brilliance iCT CT scanner (256-slice; Philips Healthcare, Cleveland, OH, USA) were used to perform the CT scans in our study. Furthermore, the acquisition parameters were listed as follows: tube voltage, 100–120 kVp; matrix, 512 × 512; tube current, 200–320 mAs; and slice thickness, 0.625–5.000 mm. Arterial phase (25–38 s), portal vein phase (55–85 s), and delay phase (120–180 s) CT scans were performed after the injection of a contrast agent (80–100 ml) into the vein of the forearm (3.0 ml/s).

### 2.4 Process

**Figure 1:**
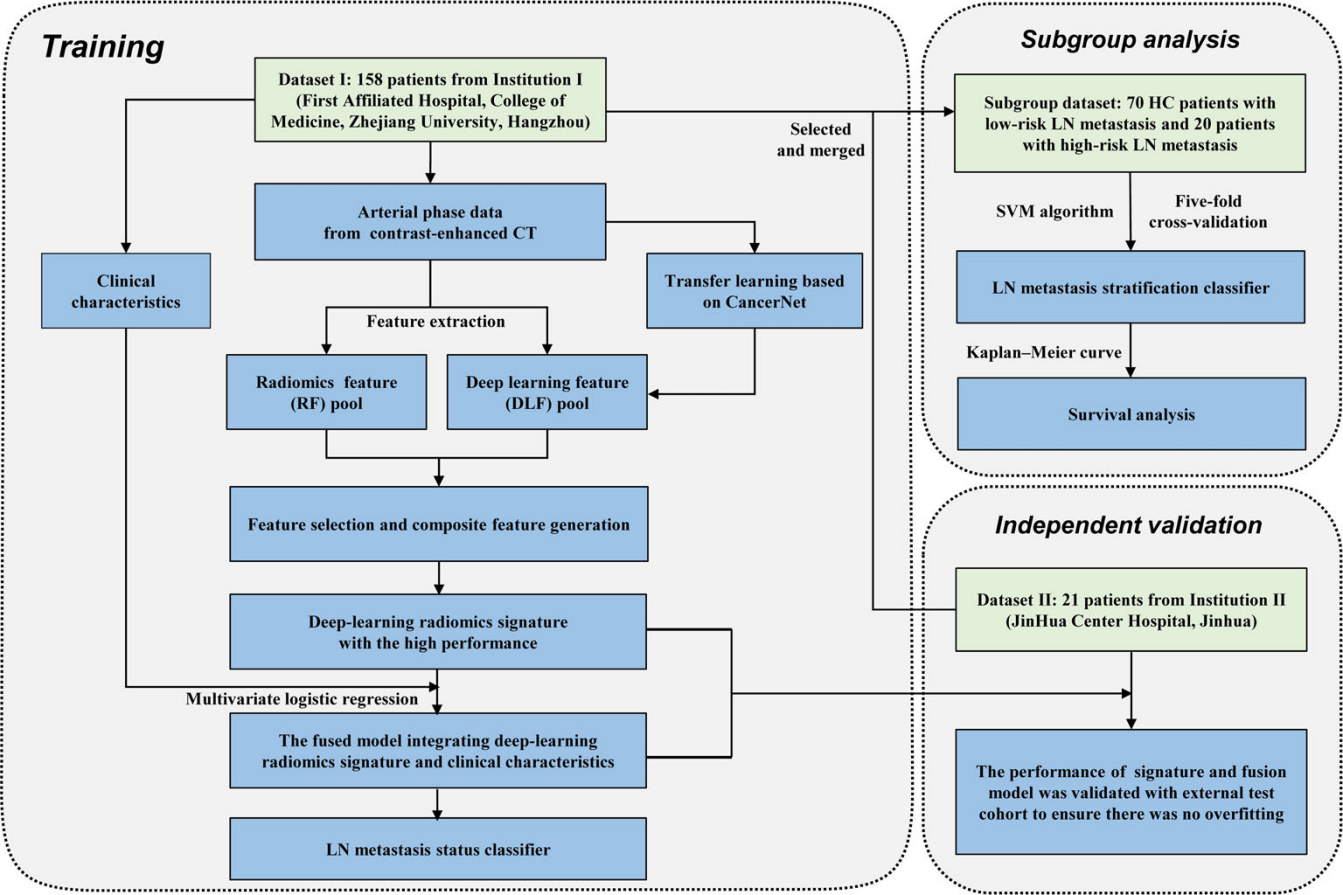
Flowchart of this two-center study.

The workflow of this study is shown in **Figure 1**. Firstly, imaging data from the two institutions were preprocessed and the radiomic features (RadFs) were extracted. Then, a convolutional neural network (CNN) structure was trained with homologous breast cancer data and the method of transfer learning. Secondly, deep learning features (DLFs) were obtained from the fully connected layer of the fine-tuned CNN structure. Thirdly, a deep learning radiomics signature (DLRS) was built with a support vector machine (SVM). An LN metastasis status classifier was proposed by integrating the clinical factors and DLRS. An LN metastasis stratification classifier was also built according to the results of subgroup analysis.

## 3 Theory and Calculation

### 3.1 Tumor Region Segmentation

ITK-SNAP was used to segment the regions of interest (ROIs) according to the maximum cross-section layer of the arterial phase images of lesions. Segmentation was firstly obtained by an experienced radiologist. Then, the result was reviewed by other radiologists to guarantee the reliability of the ROIs. The usage of ITK-SNAP is briefly introduced in *Method S1*.

### 3.2 Feature Extraction

#### 3.2.1 RadF Extraction

Several preprocesses were implemented before feature extraction to improve the stability of the extracted features (17, 18) (see *Method S2*). Four types of RadFs were extracted and used, namely, intensity-based histogram features, texture features, wavelet features, and local binary pattern (LBP) features (see *Method S3*). Then, the imaging data of 38 patients randomly selected from the dataset were re-segmented by radiologists and the intraclass correlation efficient (ICC) calculated and used as an indicator verifying the robustness of all extracted RadFs. RadFs with an ICC > 0.75 were filtered out and used for model construction.

#### 3.2.2 Deep Learning Feature Extraction

**Figure 2:**
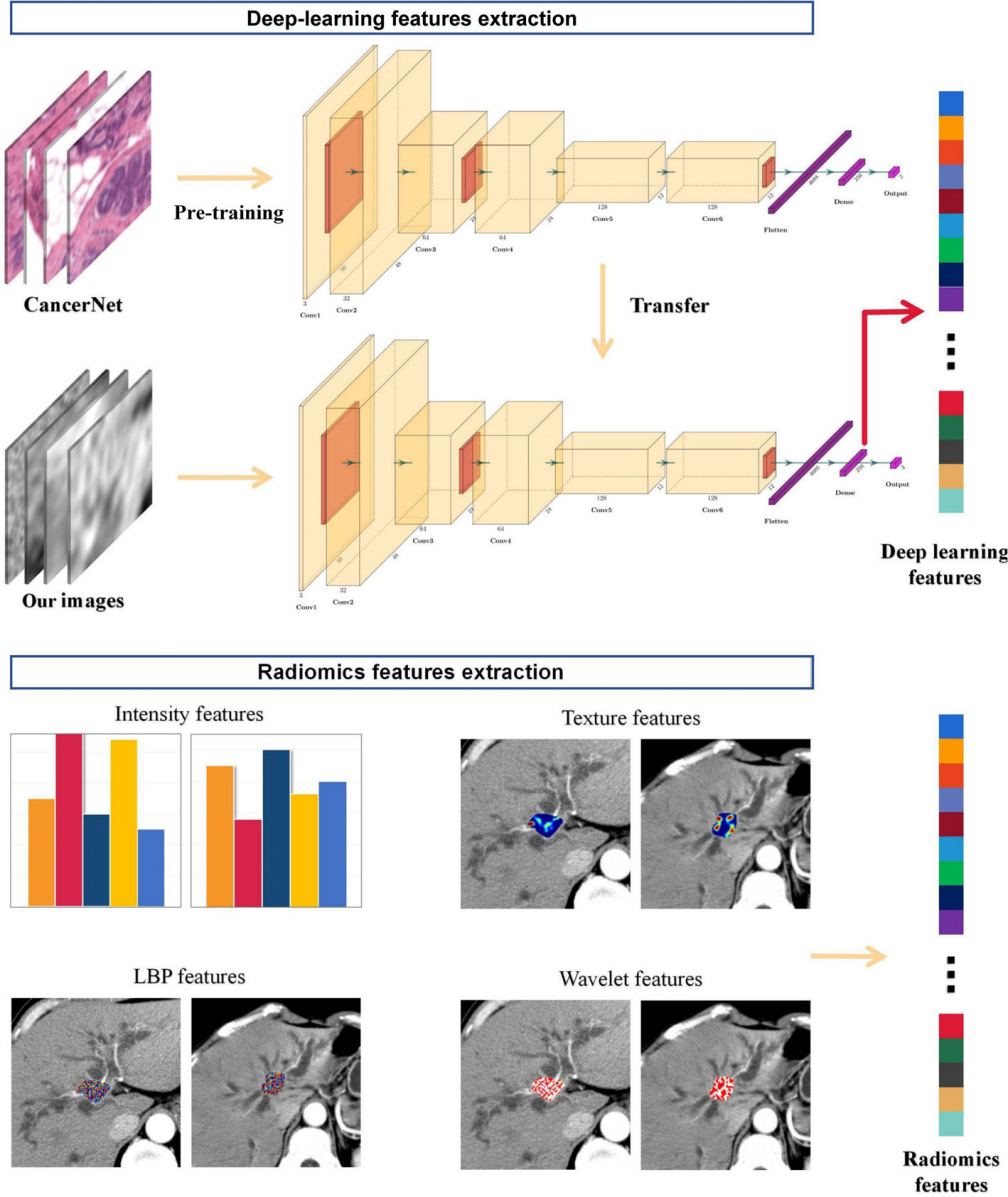
Workflow of feature extraction in hilar cholangiocarcinoma (HC) patients.

DLFs were also examined. Due to the limitation of patient population, transfer learning was considered as the optimal method to train the CNN structure for our own dataset. Firstly, the CNN structure was pre-trained with an open breast cancer dataset from Kaggle (19). Then, for weight transfer, the pre-trained CNN structure was further fine-tuned with our own dataset. The data from institution I were randomly divided at a ratio of 80%:20% for training and internal validation. Model performance was evaluated with the classification accuracy on the internal validation set. The optimal structure from five CNN structures was selected according to the classification accuracy. Finally, DLFs were extracted from the fully connected layer of the optimal structure. More details on DLF extraction are described in *Process*. The flowchart of feature extraction is shown in **Figure 2**.

### 3.3 Feature Selection and Feature Combination

Before the training of classifiers, features were selected and combined to reduce the information redundancy in the feature set and to add additional valuable information by combining the existing features. Firstly, radiomic features with low variance were gradually removed to get rid of features with small value changes. Then, correlation-based feature selection was applied to both RadFs and DLFs in order to discard redundant features. To simplify the learning procedure of the classifier and add valuable information to the feature set, a symbolic regression (SR) based on the genetic algorithm was implemented to reasonably combine the original features into new features (20). The outputs of SR were joined together as composite features (CFs) in the feature set. More details on building the composite features are available in *Method S5*. Finally, fivefold cross-validation was used to screen out the beneficial features for model construction.

### 3.4 Construction of LN Metastasis Status Classifier

With the features screened out, the DLRS was built using a fivefold validation-based SVM algorithm. The performance of the DLRS was evaluated *via* receiver operating characteristic curve (ROC) and area under the ROC curve (AUC). Then, a fusion model integrating DLRS and the clinical characteristics through multivariate logistic regression was proposed. Integrated discrimination improvement (IDI) was used to assess the significance of improvement on the classification efficiency between DLRS and RS. Decision curve analysis was conducted and considered as an important indicator showing the net benefit of each proposed classifier under different thresholds. Finally, the fusion model was selected as the LN metastasis status classifier and a diagnostic nomogram was plotted. Also, the radiologist-reported prediction and the fusion model outcomes were compared to determine whether the fusion model can help radiologists in clinical practice.

### 3.5 Construction of LN Metastasis Stratification Classifier

We think that by fine-tuning the parameters of the LN metastasis status classifier, a new classifier can be made to stratify the risk of patients with LN metastasis (N1 *vs.* N2), which is a potential biomarker in predicting patients’ overall survival (OS). Before training, the data from institutions I and II were merged because there was no N2 patient from institution II. Patients without LN metastasis were removed from the dataset. Then, the optimal CNN structure selected when constructing the LN metastasis status classifier was re-trained with the remaining patients (80% of 90 patients for training and 20% for validation). With the new DLFs extracted from the fine-tuned CNN structure and the RadFs, a new DLRS was obtained by SVM. Furthermore, the LN metastasis stratification classifier was constructed with fivefold cross-validation conducted for model evaluation. Considering there was imbalance between the population of cases and controls, area under the precision-recall curve (AUPRC) was also implemented to offer more confidence in the performance of the stratification classifier.

### 3.6 Statistical Analysis

Statistical results were obtained with R 3.6 and Python 3.7. Moreover, IDI and decision curve analysis (DCA) were performed with the packages PredictABEL and decisionCurve in R, respectively. The ROC and AUC results were acquired with the package pROC in R. Composite features were generated with the package gplearn in Python. All CNN structures were trained with TensorFlow.

## 4 Results

### 4.1 Clinical Characteristics of Patients

Seven clinical characteristics were collected, namely, sex, CEA level, CA 19-9 level, age, maximum tumor diameter, clinical stage, and CT-reported LN status. Except for the CEA level, there was no significant difference in the characteristics between the two groups. The statistical results of the seven characteristics in different datasets and different metastatic groups are shown in **Table 1** and **Supplementary Table S1**, respectively.

**Table 1:** Clinical characteristics of patients with hilar cholangiocarcinoma (HC) in different datasets.

Clinical characteristic	All patients (*n* = 179)	Training cohort (*n* = 158)	External test cohort (*n* = 21)	*p*-value (training *vs.* external)
**Sex**				0.475
Male	110	99 (63%)	11 (52%)	
Female	69	59 (37%)	10 (48%)	
**Age (years), range**	61.7 ± 8.9	61.3 ± 9.0	64.6 ± 7.2	0.566
**Maximum tumor diameter (cm), range**	2.4 ± 0.9	2.4 ± 1.0	1.9 ± 0.6	0.055
**CEA level**				<0.001
Positive	69	49 (31%)	20 (95%)	
Negative	110	109 (69%)	1 (5%)	
**CA 19-9 level**				0.428
Positive	133	119 (75%)	14 (67%)	
Negative	46	39 (25%)	7 (33%)	
**Clinical stage**				0.815
I/II	103	90 (57%)	13 (62%)	
III/IV	76	68 (43%)	8 (38%)	
**CT-reported LN status**				0.593
Positive	43	37 (23%)	6 (29%)	
Negative	136	121 (77%)	15 (71%)	
**LN metastasis**				0.496
Positive	90	81 (51%)	9 (43%)	
Negative	89	77 (49%)	12 (57%)	

Six patients were not included because of incomplete clinical data. The threshold values for distinguishing the levels (positive or negative) of CEA and CA 19-9 were 5.0 ng/ml and 37.0 U/ml, respectively. The statistical results of continuous variables were obtained based on a two-sided Mann–Whitney U test. The statistical results of categorical variables were acquired using a two-sided chi-squared test.

CEA, preoperative plasma carcinoembryonic antigen; CA 19-9, carbohydrate antigen 19-9; LN, lymph node; CT, computed tomography.

### 4.2 Features

In total, 1,067 quantitative RadFs were obtained from the ROIs of the CT data, including 7 first-order histogram features, 848 wavelet features, 159 LBP features, and 53 texture features. Five of the 53 texture features from neighborhood gray-tone difference matrix (NGTDM), 13 from gray-level size zone matrix (GLSZM), 13 from gray-level run length matrix (GLRLM), and 22 from gray-level co-occurrence matrix (GLCM) were extracted. The specific extraction of RadFs is listed in **Supplementary Table S2**.

**Figure 3:**
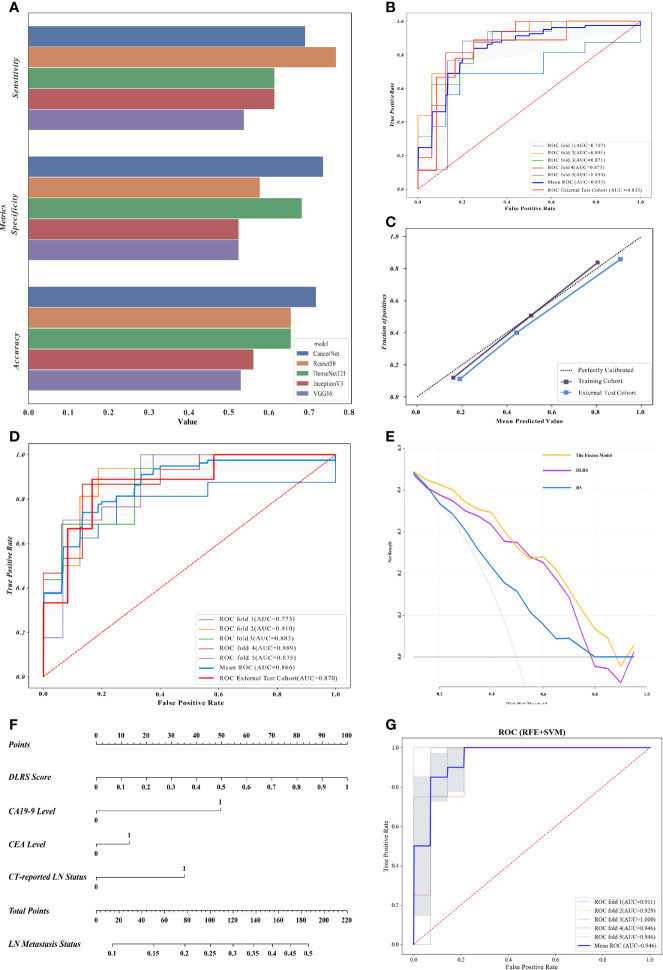
Deep learning radiomics nomograms and evaluation of the proposed classifiers. **(A)** Evaluation of five convolutional neural network (CNN) structures in the internal validation cohort, in which CancerNet showed optimal performance. **(B)** Receiver operating characteristics (ROCs) of the support vector machine (SVM)-based deep learning radiomics signature (DLRS) in the construction of the lymph node (LN) metastasis status classifier. Areas under the ROC curve (AUCs) are listed in the lower right corner. **(C)** Calibration curves of DLRS indicating that the predicted outcomes coordinated well with the real LN status. **(D)** ROCs of the fusion model integrating DLRS, the carbohydrate antigen 19-9 (CA 19-9) level, the carcinoembryonic antigen (CEA) level, and the computed tomography (CT)-reported LN status. **(E)** Decision curve analysis (DCA) showing that the fusion model is optimal for LN metastasis status assessment. **(F)** Deep learning radiomics nomogram based on the DLRS score, CEA level, CA 19-9 level, and the CT-reported LN status. **(G)** ROCs of the LN metastasis stratification classifier.

For DLF extraction, five CNN structures (CancerNet, ResNet50, VGG16, IncerptionV3, and DenseNet121) were considered. According to the performance of the five CNN structures, CancerNet was selected as the optimal network structure for feature extraction, with accuracies of 0.719 (status classification) and 0.722 (stratification classification) in the internal validation set (**Figure 3A**). This procedure was conjectured from the result that the high performance of CancerNet should be ascribed to its lower complexity compared with the other structures, such as VGG16 and ResNet50. A CNN structure with suitable complexity is easier to train and against overfitting when the population of images is limited.

The structure of CancerNet, a lightweight CNN network specifically designed for breast cancer classification, is well described in **Supplementary Table S3**. Layer Dense_2, the first fully connected layer before the output layers, was used as the DLF generator considering the structure of CancerNet. The CT images awaiting identification were sent to the fine-tuned CancerNet, while the 256 features inside layer Dense_2 were extracted and saved as the DLF features. The procedure for DLF extraction was conducted twice since there were two classification tasks in our research: one is the LN metastasis status classification and the other is the LN metastasis stratification. Visualization of the CNN intermediate layer is presented as an example in **Supplementary Figure S2**.

### 4.3 Feature Selection and Combination

Firstly, according to the ICC results, 510 RadFs passed the examination and were included in the feature set. Then, these 510 RadFs along with 256 DLFs were further filtered to obtain the feature sets that were useful for classifier training. For the LN metastasis status classifier, 527 features were removed because of low variance or high correlation. Fifty composite features were generated in accordance with the 239 remaining features. Recursive feature elimination and cross-validation (RFECV) selected a subset that included 30 features to train and test the LN metastasis status classifier. For LN metastasis stratification classification, 273 features were removed because of low variance or high correlation. After the composite features were generated, 26 features were selected by RFECV. The features finally included for status classifier modeling and stratification classification are available in **Supplementary Tables S4** and **pS5**. Two different methods were realized to visualize the difference of the selected features between the case group and the control group (**Supplementary Figures S3, S4**). According to the results, the difference was significant between the two groups and a nonlinear relationship between the features and prediction target was found.

### 4.3 Construction of the LN Metastasis Status Classifier

The SVM-based DLRS performed well with average AUCs of 0.835 in the training cohort and 0.833 (95% CI = 0.641–1.000) in the external test cohort. The result of the fivefold cross-validation showed no significant gap on the AUC of each fold, which indicated no significant overfitting. The ROCs of the SVM-based DLRS are shown in **Figure 3B**. The DLRS-predicted outcomes coordinated well with the real LN status in both the training and external test cohorts (**Figure 3C**).

A combination of the DLRS and clinical characteristics was determined according to Akaike information criterion (AIC). Finally, three clinical characteristics, i.e., the CEA level, CA 19-9 level, and CT-reported LN status, were selected for the construction of the fusion model (AIC = −289.23). Details are shown in *Formula S1*. The assembly obtained excellent AUCs of 0.866 in the training cohort and 0.870 in the external test cohort (**Figure 3D**). More assessment criteria are available in **Table 2**. Correlation analysis of the DLRS and clinical characteristics is shown in **Supplementary Figure S5**.

**Table 2:** More assessment criteria for the deep learning radiomics signature (DLRS) and the fusion model predicting the status of lymph node (LN) metastasis.

Model	Cohort	Criterion			
		Recall	Precision	F1 score	Accuracy
DLRS	Training	0.766	0.740	0.753	0.752
	External test	0.778	0.778	0.778	0.810
Fusion model	Training	0.806	0.754	0.779	0.770
	External test	0.667	0.857	0.750	0.810

Each criterion of the training cohort was obtained by averaging the criterion values fivefold.

To prove the value of the DLFs, a radiomics signature was constructed with the same method and RadFs. For the training cohort, the comparison of the DLRS and RS showed that the average AUC was improved by 15.8% (0.835 *vs.* 0.721) after consideration of the DLFs. IDI revealed that DLRS outperformed RS in distinguishing the present and absent cases (IDI = 0.1529, 95% CI = 0.0864–0.2194, *p* < 1e−5). For the external test cohort, the AUC was improved by 16.8% (0.833 *vs.* 0.713), while the confidence of improvement was insignificant, which was due to the small population of patients in the external test cohort (IDI = 0.1758, 95% CI = −0.0621 to 0.4137, *p* = 0.1476). It is assured that the DLFs contained additional information that helped improve the accuracy of classification. A clinical model was also constructed to confirm the special contribution of DLRS to the fusion model (**Supplementary Figure S6**). The fusion model outperformed both RS and DLRS in terms of net benefit and, thus, should be the optimal one among the three models (**Figure 3E**). The nomogram for LN metastasis status (**Figure 3F**) is beneficial for clinical application of the status classifier.

To validate the performance of the LN metastasis status classifier in clinical practice, we compared its outcomes with assessment using the naked eye (**Supplementary Figure S7**). The accuracy of the status classifier was significantly higher than that of the prediction given by radiologists in both the training cohort (14.6% higher) and the external test cohort (14.3% higher). The status classifier did much better than did the radiologists when checking the LN metastasis cases, which indicated the potential value of the status classifier in clinical application.

### 4.4 Construction of the LN Metastasis Stratification Classifier

Firstly, the SVM-based DLRS constructed for stratification classification performed well, with an average AUC of 0.946 (**Figure 3G**). Then, a fusion model was developed based on a clinical characteristic (CT-reported LN status) and the probability score obtained from DLRS. However, the accuracy was not significantly improved after the clinical characteristic was added in. Therefore, DLRS was selected as the ideal LN metastasis stratification classifier. The precision-recall (PR) curve was made and the AUPRC of the proposed classifier reached 0.801 (**Supplementary Figure S8**).

## 5 Discussion

We utilized two deep learning models to preoperatively predict the LN stage of HC patients. For evaluation of the LN status, the DLRS established by SVM had excellent performance, with AUCs of 0.835 and 0.833 for the training cohort and the external test cohort, respectively. Meanwhile, the LN metastasis status classifier incorporating DLRS and clinical characteristics showed AUCs of 0.866 for the training cohort and 0.870 for the external test cohort. For evaluation of the LN metastasis risk, an SVM-based DLRS was proposed, which performed well in predicting the risk stratification of LN metastasis, with an average AUC of 0.946. The results above confirmed that the LN staging prediction classifier will be effective for preoperative regional LN staging of HC patients.

Deep learning has received remarkable attention in recent years and achieved state-of-the-art performance in specific clinical applications (21). In particular, CNN can exceed visual evaluation to recognize complex image structural patterns in a data-driven manner, and it has been used in automatic detection, classification, staging, and volume segmentation (22). In previous studies, the learning of CNN structures based on a tremendous number of clinical images of skin lesions, optical coherence tomography images of retinal diseases, or endoscopic images of ulcerative colitis has achieved high-accuracy classification prediction (23–26). CNN has also been applied to ultrasound, X-ray film, and CT for the classification of child pneumonia and liver fibrosis staging (25, 27, 28). Different from the above deep learning research with adequate training data, a single-CNN research on liver lesions based on hundreds of imaging data was not outstanding (21). In our study, CancerNet can predict the status of LN metastasis with an accuracy of 0.719, which is a significant improvement compared with assessment using the naked eye (0.614). This result indicates that CNN is one of the potential solutions to improving the efficiency and accuracy of preoperative LN stage assessment.

Previous studies have proven that incorporating deep learning feature sets with traditional radiomic feature sets is a promising method for tumor phenotype evaluation. To predict the LN metastasis risk in gastric cancer, DLFs based on transfer learning were applied to construct a deep learning radiomics nomogram and showed high predictive value for individual evaluation, with C-indexes of 0.821 (primary cohort), 0.797 (external validation cohort), and 0.822 (international validation cohort) (29). Similarly, the RadFs and DLFs acquired by CNN transferred from ImageNet were combined to predict the axillary LN status of early-stage breast cancer, with an AUC of 0.902 (30). Although the predictive ability of deep learning has been proven, almost no relevant research has been conducted to predict the LN stage of HC patients since a considerable proportion of patients with advanced HC cannot receive surgical treatment. We gathered imaging data from two centers. Transfer learning was used on a lightweight CNN structure against the risk of overfitting. Then, the fine-tuned CNN structure was used as a feature generator to extract DLFs. Along with the traditional RadFs, deep learning radiomics-based classifiers were built and were proven to be effective in LN staging assessment, with average AUCs of 0.870 and 0.946. Meanwhile, we further dug the values of the proposed classifiers in clinical application. It was confirmed that the LN status classifier greatly outperformed the radiologists in terms of checking the LN metastasis cases and will offer suggestions on decision-making.

The preoperative plasma CA 19-9 level as a clinical predictor was included in our combination nomograms for LN staging in HC. In previous studies, the CA 19-9 level as a predictor played an important role in predicting intrahepatic cholangiocarcinoma stage (31, 32). Furthermore, the fusion model incorporating the CA 19-9 level achieved better predictive performance than did the RS based only on imaging data (16, 33). However, in a group of unclassified cholangiocarcinomas, the plasma CA 19-9 level was poorly predictive as an independent predictor in models of LN status prediction (15). Unfortunately, the effect of the CA 19-9 level was not explored in a recent magnetic resonance imaging-based radiomics study for LN status prediction of extrahepatic cholangiocarcinoma (34). In our study, the CA 19-9 level was included in our nomograms for the LN staging of HC patients and improved the prediction efficiency of the models.

Our deep learning radiomics nomograms will facilitate individualized comprehensive treatment strategies for HC. In current clinical practice, neither the short diameter of LNs nor heterogeneity enhancement can effectively evaluate the LN status of HC patients. In contrast to these traditional imaging characteristics, our models constructed using RadFs and DLFs showed outstanding performance in predicting the LN stage of HC patients. Subsequently, patients with LN metastasis will benefit from individualized LN dissection. Due to the poor therapeutic effect of surgery alone for HC, traditional chemotherapy and radiation are needed to improve the prognosis of patients with LN metastasis (35). A recent meta-analysis has demonstrated that neoadjuvant chemotherapy has potential benefits for HC patients (36). Therefore, neoadjuvant chemotherapy should be attempted to improve the prognosis of HC patients with high-risk LN metastasis.

This deep learning radiomics study has a few limitations. Firstly, although HC is a common primary malignancy of the liver, the cases enrolled here were limited because of the difficulty in image acquisition. Secondly, we used 2D images for deep learning and extraction of the radiomic features. Thirdly, we did not include genetic data related to LN metastasis because our goal was to construct appropriate deep learning radiomics models for the LN staging of HC patients. Because of these limitations, a future plan is made to continuously promote the prediction efficiency and clinical application of the proposed classifiers. International datasets will be included to expand the sample size. Then, a 3D automatic segmentation will be developed to take the burden off radiologists. The construction of a multi-class CNN structure will also be considered. Finally, we will incorporate more proven clinical predictors into the classifiers to further improve the classification accuracy of preoperative assessment.

In conclusion, noninvasive preoperative prediction models were developed for regional LN staging of HC patients, which showed excellent prediction performance. Our predictive models will be useful for identifying individual regional LN stage in order to guide personalized therapeutic schedules.

## 6 Conclusion

Two deep learning-based classifiers derived from computed tomography images performed well in predicting the LN stage of HC patients. The proposed models are promising and reliable evaluation tools and will improve the decision-making on differential diagnosis and the prediction of overall survival in HC patients.

## Data Availability Statement

The raw data supporting the conclusions of this article will be made available by the authors, without undue reservation.

## Ethics Statement

The Institutional Review Boards of both The First Affiliated Hospital, School of Medicine, Zhejiang University (institution I) and JinHua Center Hospital (institution II) approved this study and waived the requirement of informed consent from patients.

## Author Contributions

LW, YD, and WL were responsible for the study conception and design. JS, SR, WT, SC, and WX contributed to data analysis and interpretation. PW and YC wrote, reviewed, and edited the manuscript. All authors contributed to the article and approved the submitted version.

## Funding

This work was supported by the Natural Science Foundation of China (NSFC grant no. 81971686), the National Key Research and Development Program of China (grant no. 2018YFE0183900), the Scientific Research Fund of Zhejiang Provincial Education Department (grant no. Y202045565), and Jinhua City Science and Technology Research Project (grant no. 2021-4-168).

## Conflict of Interest

The authors declare that the research was conducted in the absence of any commercial or financial relationships that could be construed as a potential conflict of interest.

## Publisher’s Note

All claims expressed in this article are solely those of the authors and do not necessarily represent those of their affiliated organizations, or those of the publisher, the editors and the reviewers. Any product that may be evaluated in this article, or claim that may be made by its manufacturer, is not guaranteed or endorsed by the publisher.
