# Supplementary material for: Deep Learning Radiomics to Predict Regional Lymph Node Staging for Hilar Cholangiocarcinoma
Source: Front Oncol. 2021 Oct 26;11:721460. doi: 10.3389/fonc.2021.721460 (PMC8576333; doi:10.3389/fonc.2021.721460)
Supplement: Supplementary file 1 [file DataSheet_1.docx]

**Supplemental method**

**Method S1**

ITK-SNAP, a software designed for medical imaging segmentation, can outline multiple types of target lesion, such as tumor, inflammation, hemorrhage, and infarction${}^{1}$. In our research, ITK-SNAP was applied to the computed tomography images from HC patients. First, the imaging data was fed into the software. The type of images was set to arterial phase CT imaging with the dimension of figures set to be 512*512. Second, the appropriate window position was chosen to identify the boundary of the lesion more effectively and prepare for ROI drawing. Then, we focused on the maximum transverse image of tumor lesion because it is considered to be the best source for differential diagnosis. After the optimal layer and position have been selected, the radiologists have to sketch the lesion boundary with a polygon inspector. The area inside the boundary should be the ROI. During the segmentation, sharp corners should be avoided.

**Method S2**

Several preprocesses were implemented before feature extraction. Firstly, each original contrast-enhanced CT image was resampled to a 1*1 mm^2^ square, which exactly included the border of ROI. Resampling helped CNN cut out the ROI and simplified the learning task. The CNN structure focused more on learning intratumoral heterogeneity rather than locating the tumor lesion, which can improve the prediction accuracy when the population of dataset is limited. Secondly, the CT values of resampled images were quantized to 64 bins before feature extraction. Because different scales in CT values may cause discrepancy in feature values, a standardization on gray scales helps prompt the repeatability of features. Finally, to help the decision making of radiologists, a normalization on Hounsfield Unit (HU) values was applied to enhance the visualization of the lesion area.

**Method S3**

The detailed process for extracting deep-learning features related to lymph node metastasis status or stratification of HC patients:

(1) CNN structures waiting for training: Due to the limitation of the patient population, transfer learning which is suitable for our own dataset was chosen as the optimal method to train the CNN structure. According to the theory of transfer learning, the performance of the transferred CNN structure would be better if the source domain is consistent with the target domain${}^{2}$. Thus, we downloaded an open breast cancer dataset as the source domain of CNN structures${}^{3}$. Meanwhile, there is significant gap in the performances of different structures on a same resource. For this reason, we decide to select the favorable structure from five different CNN structures, including CancerNet ^4^, IncerptionV3 ^5^, DenseNet121 ^6^, ResNet50 ^7^ and VGG16 ^8^. CancerNet is a niche CNN structure constructed for the breast cancer classification. Compared with the other four traditional CNN structures that are widely used in differential diagnosis and cancer classification, the size of CancerNet is much smaller, which make it easier to train the parameters and avoid over-fitting when the population of imaging is limited.

(2) Image preprocessing for CNN structures: Firstly, the original CT images were resampled. Images were captured according to bounding boxes of ROI, and the cropped images were resized to a unified standard size according to input requirements of the different network structures. Then, to obtain the RGB images necessary for CNN input, the images with same grayscale were adopted in the R, G and B channels to synthesize the RGB images.

(3) Transfer learning: To enhance the similarity between the source and the target domain, the open breast cancer dataset was used for pre-training. After the CNN structure realized the best performance on the breast cancer dataset, all the parameters in the structure will be saved as a HDF5 file. Then, the file was loaded and used as the initial parameters of the CNN structure for our own dataset.

To avoid over-fitting, the number of changeable parameters is cut down by freezing the convolutional layers and pooling layers in the structure. Furthermore, our own dataset was used to fine-tune the parameters of fully-connected layers. The dataset from Institution I was divided into an internal training cohort and an internal validation cohort at a ratio of 4:1, which were used to fine-tune the structure and evaluate the performance respectively.

(4) Optimal parameter of CNN structure: The weights of CNN network were optimized by AdaGrad algorithm with a mini-batch size of 10. In detail, Binary cross-entropy was chosen as the objective function. With grid-searching, the learning rate was set to be 0.1 at the beginning, which was attenuated to 1*10-5. Finally, the model which indicated least accuracy loss on the internal validation cohort during the training was chosen for feature extraction. CancerNet was proved to be the best structure.

(5) Feature extraction: The superficial layer features in the network structure are general features related to source domain, and the deep layer features are more suitable for the prediction task. Furthermore, the deep-learning features in the form of vectors are extracted from one of the fully connected layers (FCLs). As for which FCL is the best DLF generator, it is recommended to choose the first FCL before the output layer in the whole structure. The FCLs are closed to the output layer and always contain the highly abstracted features, and thus are useful for the prediction task. For CancerNet, the FCL mentioned above points to layer Dense_2.

**Method S4**

Details about symbolic regression (SR):

(1) Introduction to SR: In a linear model, the target y can be represented as y=w^T x+b. However, it is difficult to find more complex relationships between features and the target even if polynomial features are added. As a supervised machine-learning algorithm, SR attempts to discover some hidden mathematical formulas between features and the target${}^{9}$. The specific implementation of SR is based on the genetic algorithm.

(2) Running SR on Python: SR is conducted on Python 3.8 with package gplearn. There are two main components of gplearn: SymbolicRegressor and SymbolicTransformer. The applications of two components are different.

SymbolicRegressor uses the formula obtained by the genetic algorithm to directly predict the target.

SymbolicTransformer is a converter that does not predict the target directly. Instead, it transforms the original features to new composite features with the operators offered by the user, which is particularly effective in the stage of feature engineering.

To simplify the learning task of the classifier and add more valuable information to the feature set, SymbolicTransformer is applied to create new features in our research.

(3) Configuration of SR: The function (operator) set of SR includes 'log', 'neg', 'inv', 'max', 'add', 'sub', 'sqrt', 'mul', 'min', 'abs', and 'div'. And the n_components of SR is set to 50, which means the output of SR includes 50 composite features selected according to the rank of spearman coefficient between the specific feature and the target.

**Supplemental figures**

**
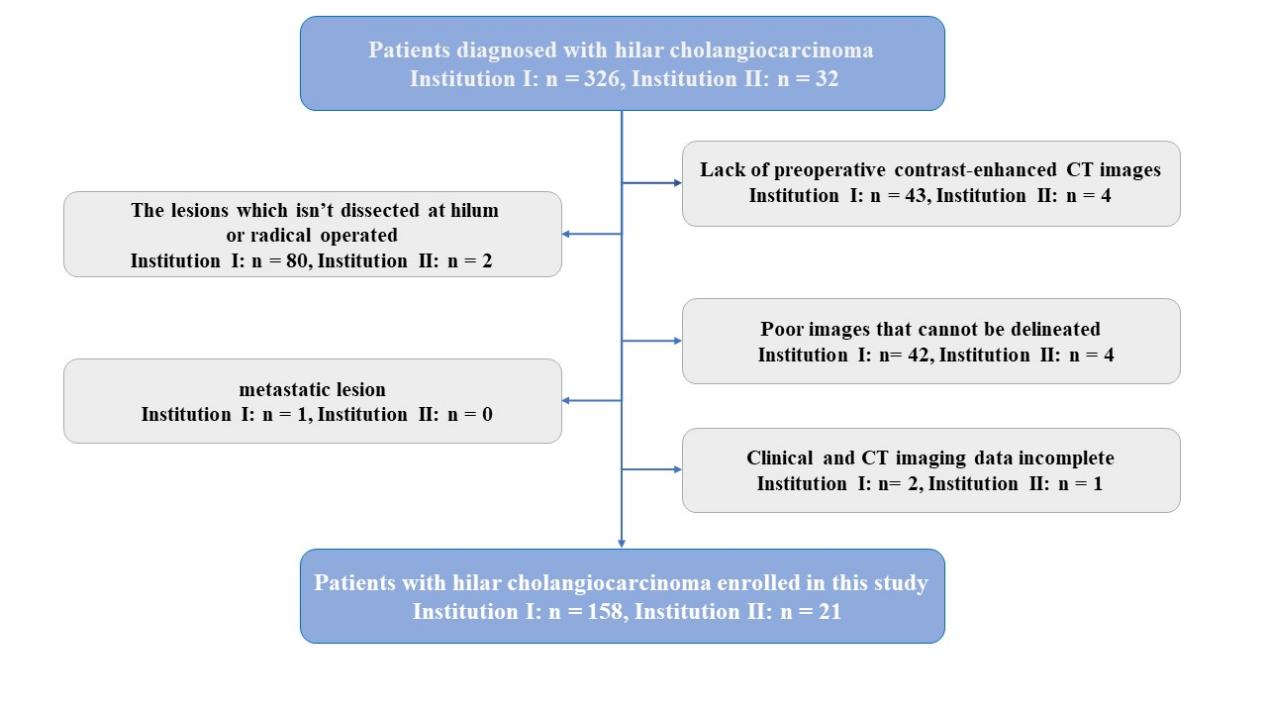
**

**Figure S1. The patient recruitment pathway.**

**
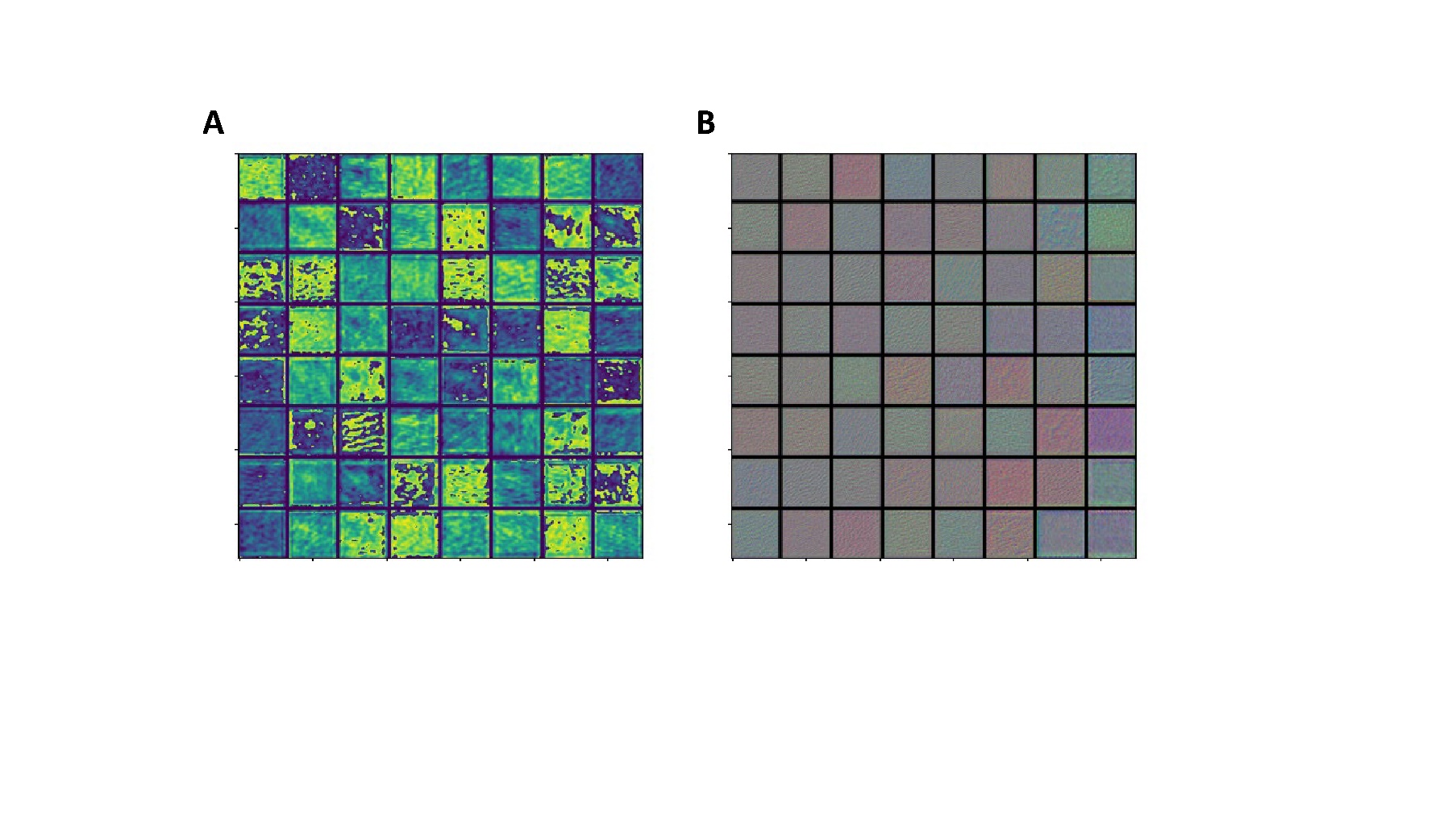
**

**Figure S2.** **The second-level convolution visualization and convolution kernel visualization of CancerNet.**

**LN metastasis status classifier**

**
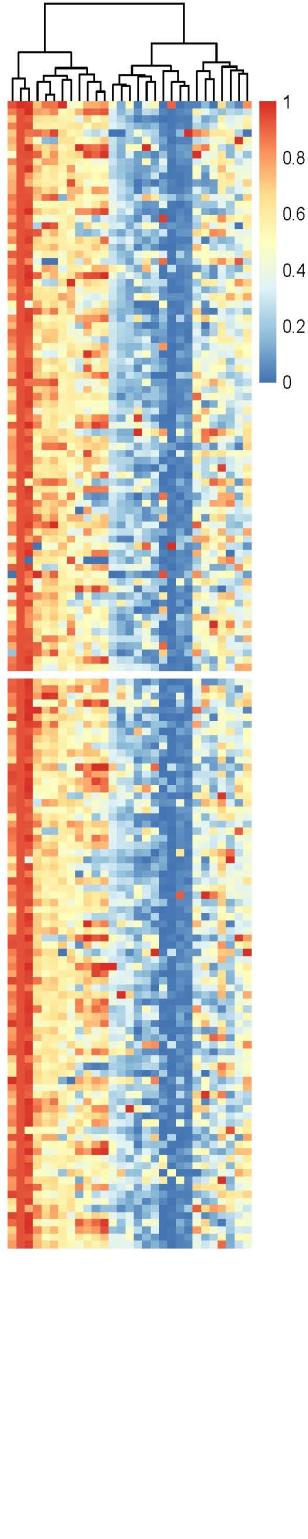
**

**LN metastasis stratification classifier**

**
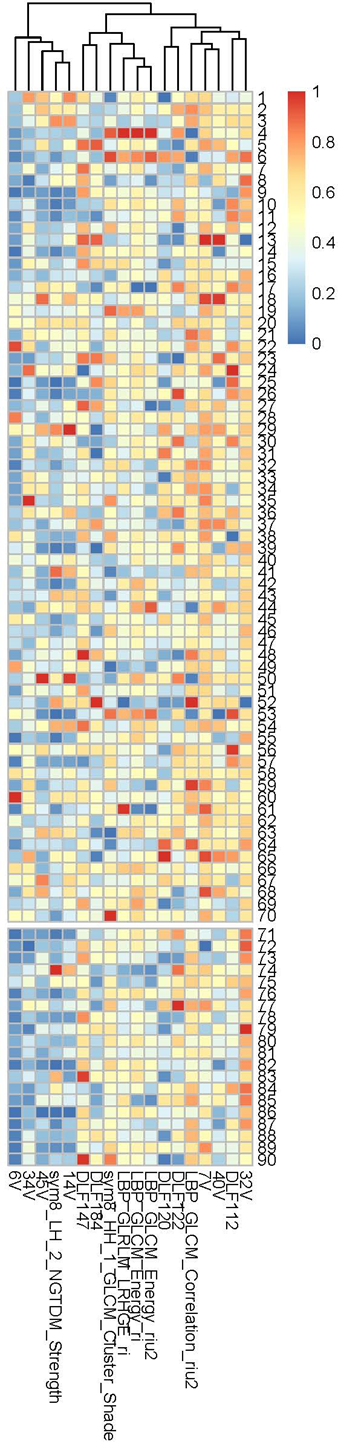
**

**
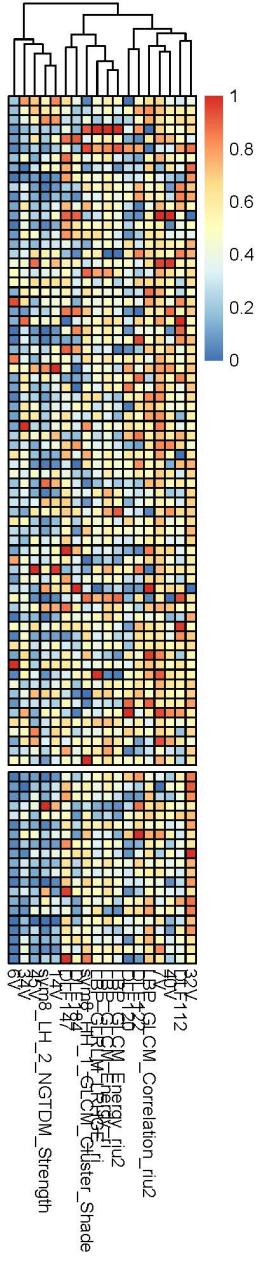
**

**Figure S3. The heatmaps of selected features that used to the construct the classifiers.** For each plot, the figure is divided into two blocks which the control group is set on the left and the case group is set on the right.

**
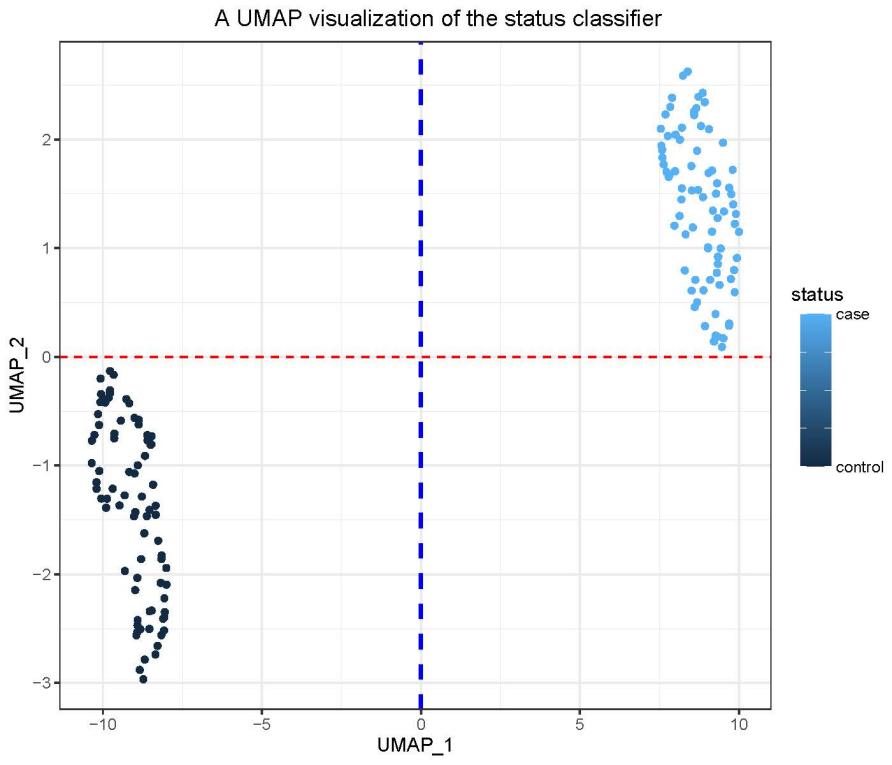
**

**
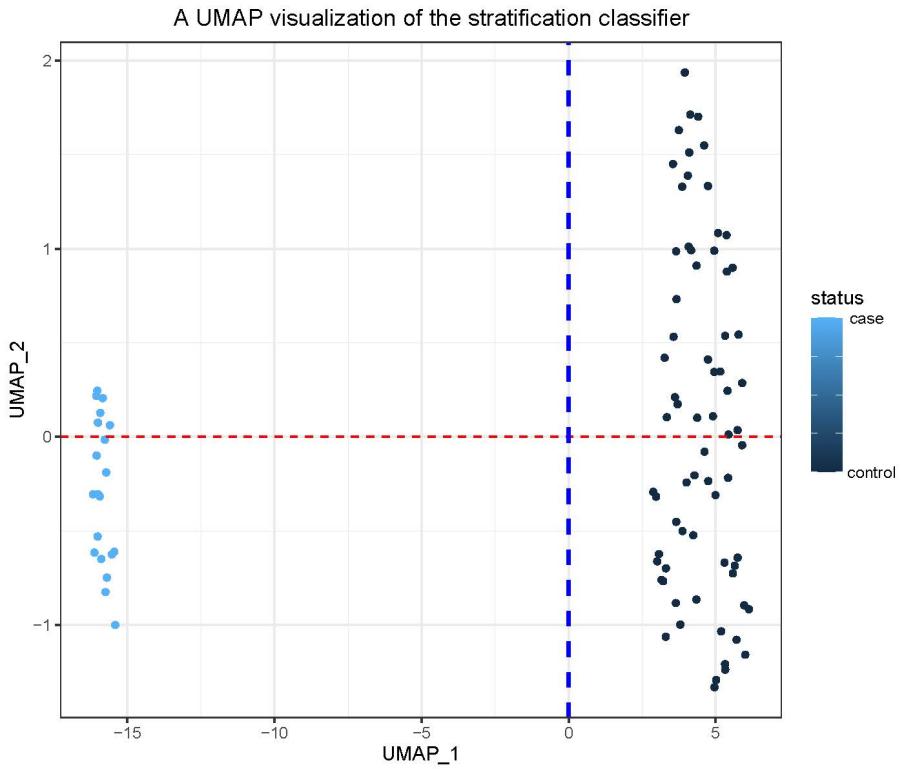
**

**Figure S4. The visualization of selected features with UMAP which indicates the selected features can well realize the classification task.** Furthermore, the results show the relationship between prediction target and features should be non-linear.

**
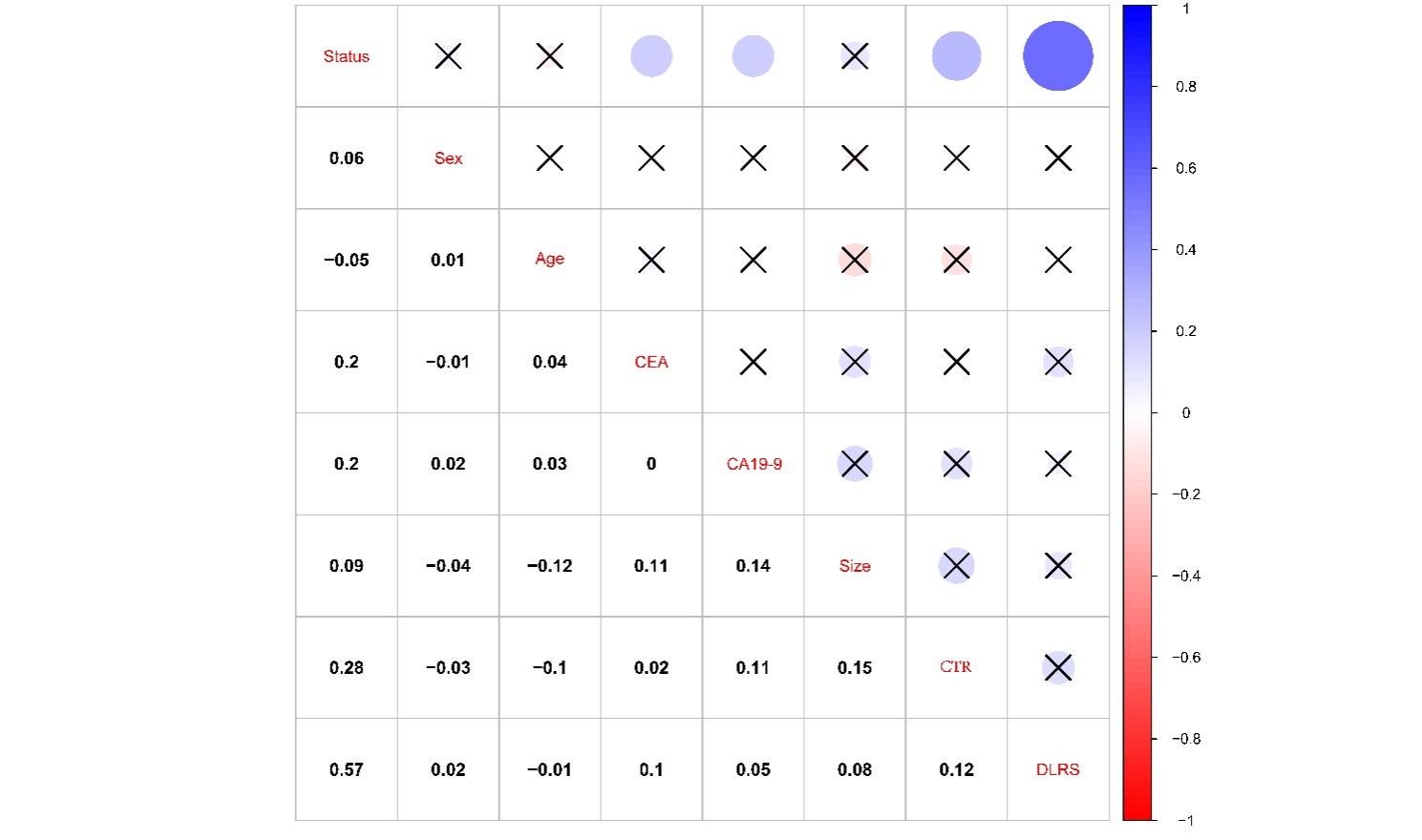
**

**Figure S5. A matrix of correlation analysis results helps to investigate the correlations among clinical characteristics, DLRS and true lymph node metastasis status.**

CEA: preoperative plasma carcinoembryonic antigen level, Size: maximum tumor diameter, CTR: computed tomography-reported lymph node status, CA19-9: preoperative plasma carbohydrate antigen 19-9 level, Status: regional lymph node metastasis status, DLRS: deep-learning radiomics signature.

**
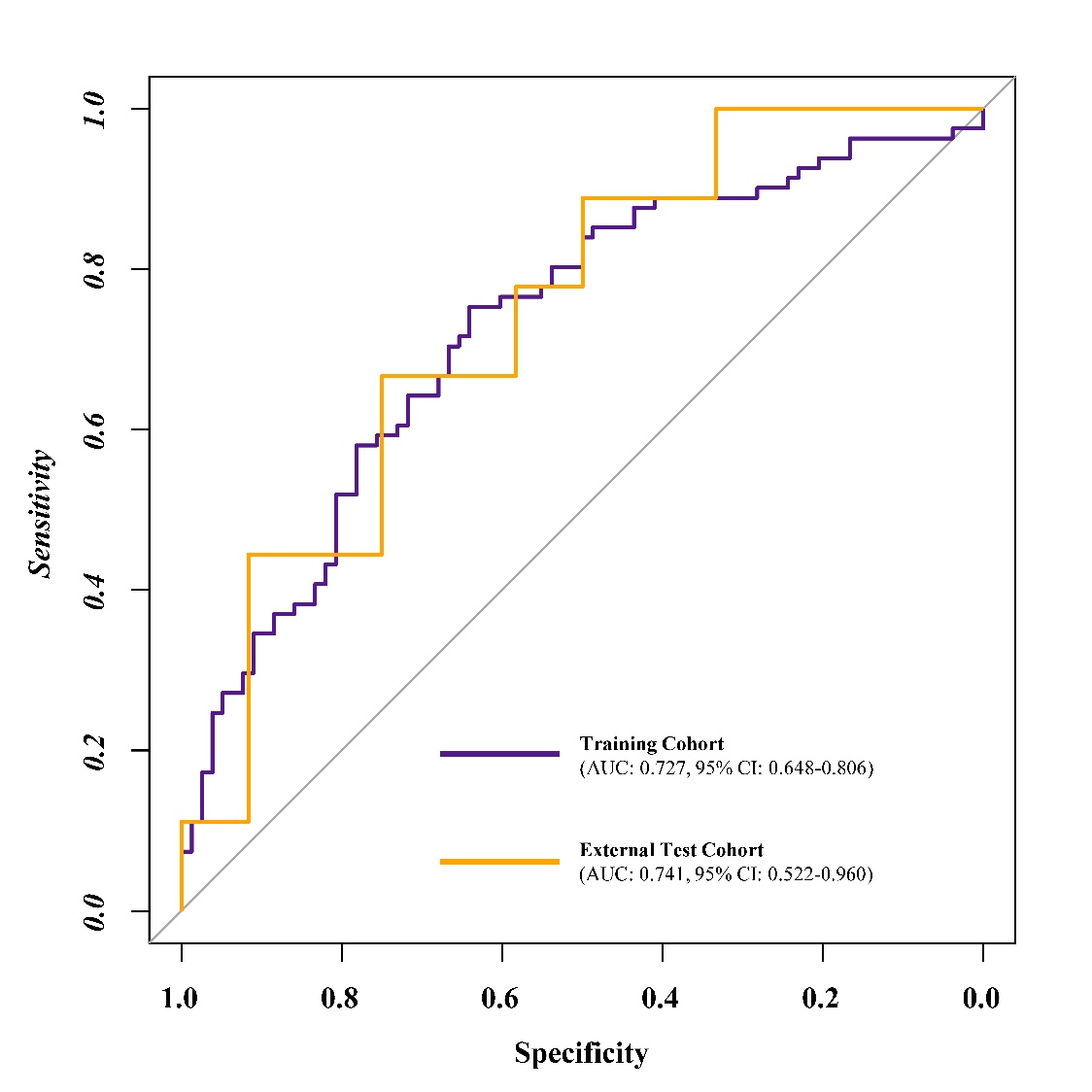
**

**Figure S6. ROCs of the clinical model in predicting the LN** **metastasis status.**

**
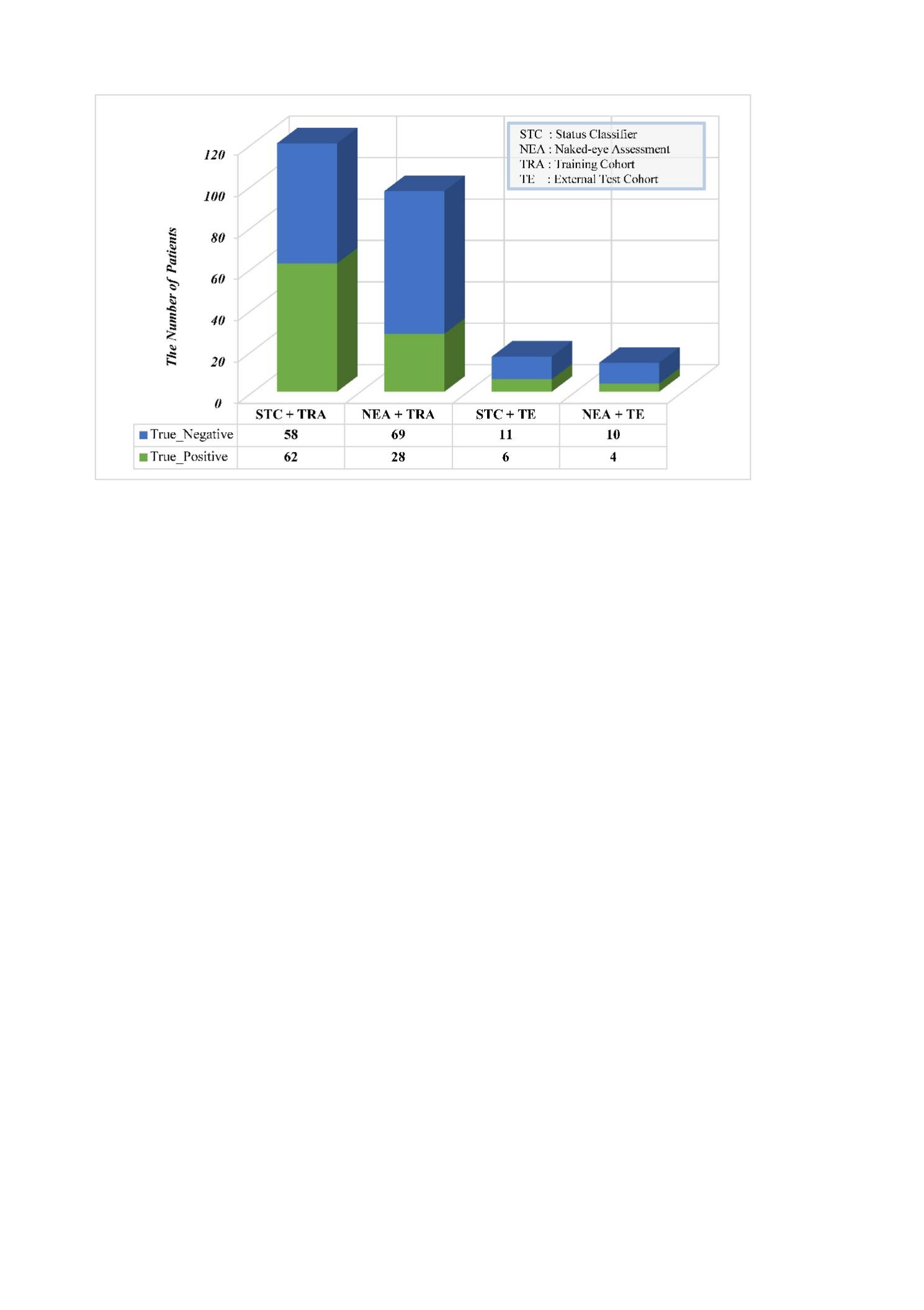
**

**Figure S7. Numbers of cases and controls correctly predicted by the LN metastasis status classifier and naked-eye assessment.**

**
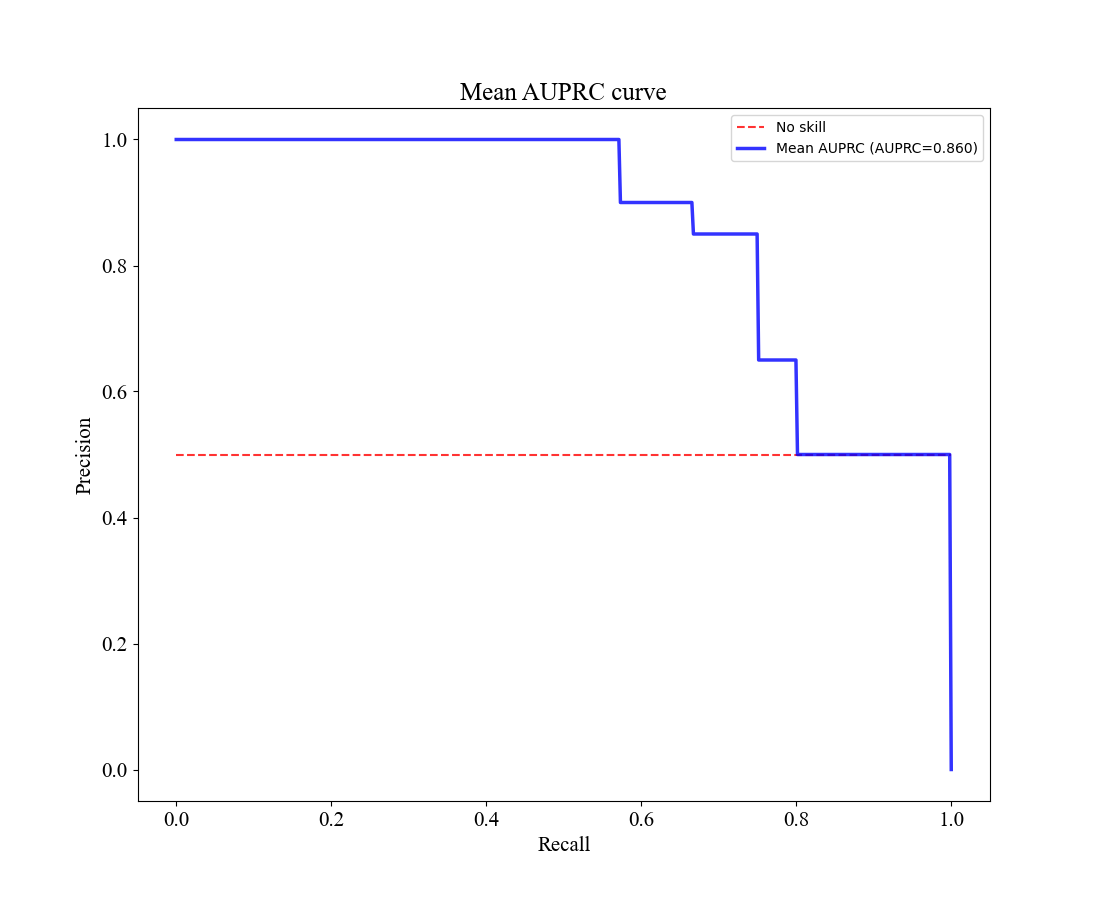
**

**Figure S8. The mean precious-recall curve of LN metastasis stratification classifier which obtains an AUPRC of 0.801.**

**Supplemental Tables**

**Table S1. Comparison of clinical characteristics between HC patients with different LN statuses.**

| **Clinical Characteristic** | **LN metastasis**  **(n = 88)** | **LN non-metastasis**  **(n = 91)** | ***p*** |
| --- | --- | --- | --- |
| **Sex** |  |  | 0.643 |
| Male | 52 (59%) | 57 (63%) |  |
| Female | 36 (41%) | 34 (37%) |  |
| **Age (years, range)** | 61.1 ± 9.5 | 62.3 ± 8.3 | 0.440 |
| **The Maximum Diameter (cm, range)** | 2.5 ± 1.0 | 2.2 ± 0.9 | 0.059 |
| **CEA** |  |  | 0.007 |
| Positive | 33 (38%) | 17 (19%) |  |
| Negative | 55 (62%) | 74 (81%) |  |
| **CA 19-9 Level** |  |  | 0.006 |
| Positive | 73 (83%) | 59 (65%) |  |
| Negative | 15 (17%) | 32 (35%) |  |
| **Clinical Stage** |  |  | 0.067 |
| Ⅰ/Ⅱ | 45 (51%) | 59 (65%) |  |
| Ⅲ/IV | 43 (49%) | 32 (35%) |  |
| **CT-reported LN Status** |  |  | <0.001 |
| Positive | 32 (36%) | 11 (12%) |  |
| Negative | 56 (64%) | 80 (88%) |  |

**Notes:** HC: hilar cholangiocarcinoma, CEA: preoperative plasma carcinoembryonic antigen and CA 19-9: carbohydrate antigen 19-9, LN: lymph node, CT: computed tomography. The values of age and the maximum diameter are shown as mean ± standard deviation. Six patients were not included because of incomplete clinical data. The statistical results of continuous variables were obtained based on a two-sided Mann–Whitney U-test. The statistical results of categorical variables were acquired through a two-sided chi-squared test.

**Table S2.** **Radiomics features used in this study.**

| **Feature Category** | **Features in detail** |
| --- | --- |
| **Histogram features** | Mean, Variance, Deviation, Skewness, Kurtosis, Energy, Entropy, |
| **Texture features** | Energy, Entropy, Dissimilarity, Contrast, Inverse difference, Correlation, Homogeneity, Autocorrelation, Cluster shade, etc. derived from GLCM.  Short Run Emphasis (SRE), Long Run Emphasis (LRE), Gray-Level Nonuniformity (GLN), Run Percentage (RP), etc. derived from GLRLM.  Small Zone Emphasis (SZE), Large Zone Emphasis (LZE), Gray-Level Nonuniformity (GLN), Zone Percentage (ZP), etc. derived from GLSZM.  Strength, Busyness, Complexity, Contrast, Coarseness derived from NGTDM. |
| **Wavelet features** | Wavelet texture features derived from images transformed in low-frequency sub-bands, horizontal high-frequency sub-bands, vertical high-frequency sub-bands, and diagonal high-frequency sub-bands at successive multiscale. |
| **LBP features** | LBP features derived from images transformed using uniform, rotation invariant and uniform rotation invariant local binary patterns. |

**Notes:** LBP, Local Binary Pattern; GLCM, Gray-Level Co-occurrence Matrix; GLRLM, Gray-Level Run-Length Matrix; GLSZM, Gray-Level Size Zone Matrix; NGTDM, Neighborhood Gray Tone Difference Matrix (the same in other tables).

**Table S3. Structure of CancerNet in detail.**

| **Layer Index** | **Layer Detail** | **Attributes of End** | **Size** |
| --- | --- | --- | --- |
| 1 | separable_conv2d_1: SeparableConv2D | input | (None, 48, 48, 3) |
|  |  | output | (None, 48, 48, 32) |
| 2 | activation_1: Activation | input | (None, 48, 48, 32) |
|  |  | output | (None, 48, 48, 32) |
| 3 | batch_normalization_1: BatchNormalization | input | (None, 48, 48, 32) |
|  |  | output | (None, 48, 48, 32) |
| 4 | max_pooling2d_1: MaxPooling2D | input | (None, 48, 48, 32) |
|  |  | output | (None, 24, 24, 32) |
| 5 | dropout_1: Dropout | input | (None, 24, 24, 32) |
|  |  | output | (None, 24, 24, 32) |
| 6 | separable_conv2d_2: SeparableConv2D | input | (None, 24, 24, 32) |
|  |  | output | (None, 24, 24, 64) |
| 7 | activation_2: Activation | input | (None, 24, 24, 64) |
|  |  | output | (None, 24, 24, 64) |
| 8 | batch_normalization_2: BatchNormalization | input | (None, 24, 24, 64) |
|  |  | output | (None, 24, 24, 64) |
| 9 | separable_conv2d_3: SeparableConv2D | input | (None, 24, 24, 64) |
|  |  | output | (None, 24, 24, 64) |
| 10 | activation_3: Activation | input | (None, 24, 24, 64) |
|  |  | output | (None, 24, 24, 64) |
| 11 | batch_normalization_3: BatchNormalization | input | (None, 24, 24, 64) |
|  |  | output | (None, 24, 24, 64) |
| 12 | max_pooling2d_2: MaxPooling2D | input | (None, 24, 24, 64) |
|  |  | output | (None, 12, 12, 64) |
| 13 | dropout_2: Dropout | input | (None, 12, 12, 64) |
|  |  | output | (None, 12, 12, 64) |
| 14 | separable_conv2d_4: SeparableConv2D | input | (None, 12, 12, 64) |
|  |  | output | (None, 12, 12,128) |
| 15 | activation_4: Activation | input | (None, 12, 12, 128) |
|  |  | output | (None, 12, 12, 128) |
| 16 | batch_normalization_4: BatchNormalization | input | (None, 12, 12, 128) |
|  |  | output | (None, 12, 12, 128) |
| 17 | separable_conv2d_5: SeparableConv2D | input | (None, 12, 12, 128) |
|  |  | output | (None, 12, 12, 128) |
| 18 | activation_5: Activation | input | (None, 12, 12, 128) |
|  |  | output | (None, 12, 12, 128) |
| 19 | batch_normalization_5: BatchNormalization | input | (None, 12, 12, 128) |
|  |  | output | (None, 12, 12, 128) |
| 20 | separable_conv2d_6: SeparableConv2D | input | (None, 12, 12, 128) |
|  |  | output | (None, 12, 12, 128) |
| 21 | activation_6: Activation | input | (None, 12, 12, 128) |
|  |  | output | (None, 12, 12, 128) |
| 22 | batch_normalization_6: BatchNormalization | input | (None, 12, 12, 128) |
|  |  | output | (None, 12, 12, 128) |
| 23 | max_pooling2d_3: MaxPooling2D | input | (None, 12, 12, 128) |
|  |  | output | (None, 6, 6, 128) |
| 24 | dropout_3: Dropout | input | (None, 6, 6, 128) |
|  |  | output | (None, 6, 6, 128) |
| 25 | flatten_1: Flatten | input | (None, 6, 6, 128) |
|  |  | output | (None, 4608) |
| 26 | dense_1: Dense | input | (None, 4608) |
|  |  | output | (None, 256) |
| 27 | activation_7: Activation | input | (None, 256) |
|  |  | output | (None, 256) |
| 28 | batch_normalization_7: BatchNormalization | input | (None, 256) |
|  |  | output | (None, 256) |
| 29 | dropout_4: Dropout | input | (None, 256) |
|  |  | output | (None, 256) |
| 30 | dense_2: Dense | input | (None, 256) |
|  |  | output | (None, 2) |
| 31 | activation_8: Activation | input | (None, 2) |
|  |  | output | (None, 2) |

**Note.** The specific number of “None” in the “Size” item represents the number of images sent to the input of structure.**Table S4. Features constructing the SVM-based deep-learning radiomics signature for the LN metastasis status classifier.**

| **Radiomics Feature** | **Composite Feature** | **Deep-learning Feature** |
| --- | --- | --- |
| GLCM_Correlation | CF No.1 | DLF No.27 |
| LL_1_NGTDM_Strength | CF No.5 | DLF No.89 |
| LL_2_GLCM_Inverse_difference | CF No.6 | DLF No.101 |
| HH_1_NGTDM_Contrast | CF No.12 | DLF No.205 |
| HL_2_NGTDM_Strength | CF No.16 | DLF No.235 |
| LH_2_GLCM_Inverse_difference | CF No.17 |  |
| LBP_GLCM_Energy_u2 | CF No.25 |  |
| LBP_GLCM_Correlation_u2 | CF No.37 |  |
| LBP_GLCM_Sum_Entropy_u2 | CF No.38 |  |
| LBP_GLSZM_LZHGE_u2 | CF No.44 |  |
| LBP_GLCM_Correlation_ri | CF No.45 |  |
| LBP_GLSZM_HGZE_ri | CF No.49 |  |
| LBP_GLRLM_GLV_riu2 |  |  |

**Notes:** DLF, Deep-Learning Feature; CF, composite feature; HGZE, High Gray-Level Zone Emphasis. The names of composite features and deep-learning features were represented by their index because they are abstract (the same in other tables).

**Table S5. Features constructing the SVM-based deep-learning radiomics signature for LN metastasis stratification classifier.**

| **Radiomics Feature** | **Composite Feature** | **Deep-learning Feature** |
| --- | --- | --- |
| LH_1_GLCM_Difference_variance | CF No.6 | DLF No.112 |
| LBP_GLCM_Energy_ri | CF No.7 | DLF No.120 |
| LBP_GLRLM_LRHGE_ri | CF No.14 | DLF No.122 |
| LBP_GLCM_Energy_riu2 | CF No.32 | DLF No.147 |
| LBP_GLCM_Correlation_riu2 | CF No.34 | DLF No.184 |
| sym8_HH_1_GLCM_Cluster_Shade | CF No.40 |  |
| sym8_LH_2_NGTDM_Strength | CF No.45 |  |
|  |  |  |

**Supplemental formula**

**Formula S1**

LN_metastasis_status = -4.1 + 5.3*DLRS_score + 0.6*CEA + 1.1*CA_19-9_level + 1.2*CT_reported_LN_status

**Notes:** LN_metastasis_status: probability of HC with lymph node metastasis; DLRS_score: outcomes of the SVM-based deep-learning radiomics signature; CA_19-9_level: carbohydrate antigen 19-9 level; CEA: preoperative plasma carcinoembryonic antigen level.

**Reference**

1. Paul Yushkevich, Guido Gerig, ITK-SNAP homepage, http://www.itksnap.org/

2. Torrey, Lisa, and Jude Shavlik, "Transfer learning." Handbook of research on machine learning applications and trends: algorithms, methods, and techniques. IGI global, 2010. 242-264.

3. Paul Mooney, Breast Histopathology Images, https://www.kaggle.com/paultimothymooney

/breast-histopathology-images

4. Adrian Rosebrock, Breast cancer classification with Keras and Deep Learning, PyImageSearch, https://www.pyimagesearch.com/2019/02/18/breast-cancer-classification-with-keras-and-deep-learning/

5. Christian Szegedy, Vincent Vanhoucke, Sergey Ioffe, Jonathon Shlens, Zbigniew Wojna, Rethinking the Inception Architecture for Computer Vision, https://arxiv.org/abs/1512.00567

6. Gao Huang, Zhuang Liu, Laurens van der Maaten, Kilian Q. Weinberger, Densely Connected Convolutional Networks, https://arxiv.org/abs/1608.06993

7. Kaiming He, Xiangyu Zhang, Shaoqing Ren, Jian Sun, Deep Residual Learning for Image Recognition, https://arxiv.org/abs/1512.03385

8. Karen Simonyan, Andrew Zisserman, Very Deep Convolutional Networks for Large-Scale Image Recognition, https://arxiv.org/abs/1409.1556

9. Trevor Stephens, Genetic Programming in python with a scikit-learn inspired API: gplearn, https://gplearn.readthedocs.io/en/stable/intro.html
